# Understanding the Reasons, Contexts and Costs of Camouflaging for Autistic Adults

**DOI:** 10.1007/s10803-018-03878-x

**Published:** 2019-01-09

**Authors:** Eilidh Cage, Zoe Troxell-Whitman

**Affiliations:** Department of Psychology, Royal Holloway, University of London, Egham Hill, Egham, TW20 0EX Surrey UK

**Keywords:** Camouflaging, Mental health, Wellbeing, Autistic adults, Gender differences

## Abstract

Camouflaging entails ‘masking’ in or ‘passing’ social situations. Research suggests camouflaging behaviours are common in autistic people, and may negatively impact mental health. To enhance understanding of camouflaging, this study examined reasons, contexts and costs of camouflaging. 262 autistic people completed measures of camouflaging behaviours, camouflaging contexts (e.g. work vs. family), camouflaging reasons (e.g. to make friends) and mental health symptoms. Findings indicated a gender difference in reasons for camouflaging, with autistic women more likely to endorse “conventional” reasons (e.g. getting by in formal settings such as work). Both camouflaging highly across contexts and ‘switching’ between camouflaging in some contexts but not in others, related to poorer mental health. These findings have implications for understanding camouflaging in autistic adults.

## Introduction

‘Camouflaging’ is a term used to describe behaviours that hide or mask aspects of oneself from others, or to ‘pass’ everyday social interactions (Hull et al. 2017). Camouflaging has been proposed as a common experience for autistic^1^ people in their navigation of the non-autistic world (Bargiela et al. 2016; Hull et al. 2017). Autism is a neurodevelopmental condition, with difficulties in social relationships and social communication, as well as heightened attention-to-detail and sensory experiences (APA 2013). Of paramount concern is the high prevalence of mental health conditions for autistic people, such as depression (Stewart et al. 2006), anxiety (Gillott and Standen 2007), social anxiety (Maddox and White 2015) and suicidal behaviour and ideation (Cassidy et al. 2014; Hirvikoski et al. 2016). One study found 79% of autistic adults had diagnosable mental health conditions (Lever and Geurts 2016). Research indicates that experiences of camouflaging could relate negatively to mental health (Bargiela et al. 2016; Cage et al. 2018a). However, more research is needed to fully understand the experience of camouflaging for autistic adults: the costs of camouflaging, the contexts in which it happens and the reasons why it happens.

Camouflaging could be costly to mental health for a number of reasons. In Hull et al.’s (2017) qualitative study examining autistic adults’ experiences, participants explained how camouflaging was both physically and mentally exhausting. Their participants reported feeling anxious and stressed after camouflaging and as though they were not being their ‘true selves’. In another qualitative study, Bargiela et al. (2016) interviewed late-diagnosed autistic women and also noted this feeling of exhaustion after camouflaging and negative impact on identity. In a quantitative study, Cage et al. (2018a) found that participants who spontaneously reported camouflaging showed greater symptoms of depression and felt less accepted by others. Camouflaging has also been found to be a risk marker for suicidality in autistic adults (Cassidy et al. 2018). These studies suggest the effort of camouflaging is costly for wellbeing and potentially has negative consequences for psychological constructions like identity.

The current study specifically aimed to examine the contexts in which camouflaging occurs in relation to the impact this may have on autistic people’s wellbeing. It could be that within different contexts, autistic people may camouflage more or less. The current study’s ideology was based on Disconnect Theory (Ragins 2008). At the core of this theory is the idea that individuals use context-specific information to inform the way they will act in that context, rather than engaging with all contexts in the same way (Ragins 2008). Accordingly, there is a “disconnection” of self-presentation and engagement between different contexts: for example, an individual may decide to openly discuss their autistic identity with friends but not with co-workers. Ragins (2008) suggested that having more disconnection may be detrimental to mental health: when an individual puts energy into keeping track of which parts of their identity are expressed in which environments, it could cause identity fragmentation, stress, anxiety and depression (Bowen and Blackmon 2003; Ragins 2008).

Ragins et al.(2007) found that disconnection between personal-life and work-life for individuals with a stigmatized sexual identity related to more stress and fear of disconnect collapse than the consequences of actually disclosing their identity in the workplace. Demonstrating the inverse effect are results from Hudson’s (2013) quantitative study, including both disabled and non-disabled participants, and examining disclosure and academic success in university. One of the primary predictors of academic and personal success was early disability disclosure (within the first year of university). Further, Hudson (2013) found that successful early-disclosing students displayed identity integration across contexts, by sharing their disability within interpersonal circles and university staff. Together, these studies demonstrate how damaging disconnection could be and how risk is minimised when individuals share disability information across life contexts. To the best of our knowledge, Disconnect Theory (Ragins 2008) has not been applied to camouflaging. It may be the case that autistic people experience a ‘camouflage disconnect’ whereby they camouflage in some but not all contexts. Based on Disconnect Theory (Ragins 2008), greater camouflage disconnection might link to reduced wellbeing in autism.

If camouflaging is detrimental to mental health, it is important to understand why camouflaging is still reported by many autistic people, therefore this study also aimed to scrutinise the possible reasons for camouflaging. It is conceivable, however, that the reasons for camouflaging may differ according to the gender of the individual. There are mixed findings around gender differences in camouflaging: one hypothesis is that camouflaging contributes to the late or misdiagnosis of autism in women (Lai et al. 2015). For example, Lai et al. (2017) found that autistic women had lower scores on the ADOS (Autism Diagnostic Observation Schedule, Lord et al. 2000) than men, reflecting ‘external presentation’, but they had comparable scores on measures of ‘internal presentation’ of autistic traits. Lai et al. (2017) argued that camouflaging occurs more for women due to greater discrepancy between ‘internal’ and ‘external’ behavioural manifestations of autism, thus linking to diagnostic differences for autistic women.

An alternative hypothesis is that both autistic men and women camouflage, but they camouflage for different reasons, partially driven by societal expectations. Some research has noted few gender differences in camouflaging (Cage et al. 2018a; Hull et al. 2017). Camouflaging could happen for many (irrespective of gender) as a response to stigmatisation, with autistic people navigating the non-autistic world and using camouflaging to do so. Here, identity processes could operate, with autistic women having to navigate their identity as autistic and as an autistic woman. The navigation of multiple identities can be thought of in terms of intersectionality (Davis 2008). Intersectionality is often used within feminist theory to deepen understanding of women’s experiences beyond gender—focusing on the intersection between gender and an individual’s other identities, such as race, sexuality or disability (Davis 2008).

Saxe (2017) argues that autistic women’s experiences can be considered within an intersectional framework—whereby autistic women are marginalised due to the male-focus that has dominated discourse about autism. For example, early definitions of autism were borne out of observations of males (Kanner 1943) and diagnostic instruments have mostly been developed based on male responses (e.g. ADOS, Lord et al. 2000). Camouflaging may occur for autistic women because society has certain expectations around what autism ‘looks’ like. Shefcyk (2015, p. 132) notes that “to be a female with an ASC [autism spectrum condition] is to be twice excluded: once from the neurotypical female population, and once again from the [autism] community”. For autistic men, camouflaging may still occur as a response to stigmatisation for being autistic, but they avoid the additional stigma of autistic womanhood. The current study therefore examined the potential reasons for camouflaging and tested whether these reasons differ between genders.

Overall, the current study aimed to enhance understanding of camouflaging by examining its reasons and contexts, and the potential costs of camouflaging for mental health. Based on the literature discussed, it was first hypothesised that camouflaging disconnects (i.e. camouflaging in some contexts but not in others) would negatively impact psychological well-being. Second, it was hypothesised that there would be gender differences in reasons for camouflaging, and that, third, age of diagnosis may interact with this, given the suggestion that camouflaging may link to mis- or late diagnosis.

## Methods

### Participants

262 autistic adults over the age of 18 took part in a survey, with a mean age of 33.62 (*SD* = 11.52; range 18 to 66). Participants self-reported diagnoses of an autism spectrum condition (ASC; 51.5%), Asperger’s Syndrome (AS; 60.3%) or Pervasive Developmental Disorder Not Otherwise Specified (PDD-NOS; 1.5%). Overlap may be driven by participants selecting both ASC and AS, given the re-categorization in the DSM-5 under the umbrella term “autism spectrum disorder” (APA 2013). Self-reported diagnoses were validated using the Ritvo Autism and Asperger Diagnostic Scale (RAADS-14; Eriksson et al. 2013) and all participants scored above the cut-off score of 14 (M = 31.70, SD = 6.36; range 14–42). Participants also self-reported other diagnoses (Table 1). The high rate of comorbidity in this sample is representative of the autism population (e.g. Lundström et al. 2015).

**Table 1:** Additional participant demographic information

	%
Age of diagnosis	
Under 18	21.2
18–34	42.8
35–64	36.0
Mental health/additional diagnoses	
Anxiety	51.9
ADHD	14.5
Bipolar	3.1
Depression	50.8
Obsessive compulsive disorder	7.6
Post-traumatic stress disorder	9.5
Social anxiety disorder	23.7
Tourette’s syndrome	1.9
Other diagnosis	18.7
Sexual identity	
Heterosexual	58.2
Gay/lesbian	9.6
Bisexual	14.9
Don’t know	6.9
Other	8.0
Prefer not to say	2.3
Ethnicity	
White	85.8
Mixed/multi-ethnic	8.4
Asian	2.7
Other	1.9
Prefer not to say	1.1
Highest level of education	
No qualifications	6.1
GCSEs or equivalent	10.8
Apprenticeship	1.1
2 + A-levels or equivalent	13.0
Undergraduate degree	28.7
Masters degree	18.8
Doctoral degree	6.1
Other qualifications	10.0
Prefer not to say	5.4
Employment status	
Employed full-time	29.6
Employed part-time	10.4
Self-employed	7.7
Unemployed	11.9
Unable to work	10.4
Retired	1.2
Student	23.8
Carer	3.8
Prefer not to say	1.2

There were 135 females (51.5%), 111 males (42.4%), and 12 participants who identified as other genders (e.g. non-binary, agender; 4.6%). Four preferred not to disclose their gender (1.5%). Further characterisation of the sample, including ethnicity, sexual identity, level of education and employment status can be seen in Table 1.

Participants were recruited through social media as well as direct contact through autism charities and organisations. All participants were provided with a study description before giving informed consent. Additionally, participants were offered participation in a prize draw. Ethical approval was obtained through the ethical procedure at Royal Holloway, University of London. All participants gave full informed consent before participating.

### Materials and Procedure

In the early stages of the research, autistic people were consulted on the relevance of the research topic for the autism community and the survey itself was reviewed by two autistic individuals. These individuals gave feedback on all items which had not been validated before (i.e. camouflaging reasons and contexts) and contributed ideas for other relevant items. An online survey was then developed using the ‘Qualtrics’ platform. Measures were presented in the order below. Data was collected between November 2017 and February 2018. On average, the survey took 20 min to complete.

#### Camouflaging Questionnaire (CAT-Q; Hull et al. 2018)

Participants completed 25-items pertaining to camouflaging behaviours, which they rated from ‘strongly disagree’ (1) to ‘strongly agree’ (7), to indicate how much they agreed the item described their social interactions. Example items included “In social situations, I feel like I’m performing rather than being myself”. A total score was created by summing responses (including reverse-scored items), and scores could range from 25 to 175. Internal consistency was good (α = 0.89).

#### Camouflaging Reasons

Participants were presented with 21 reasons for camouflaging. For each item, participants rated whether they agreed that it was a reason for camouflaging (‘strongly disagree’ (1) to ‘strongly agree’ (5)). Example reasons included “to aid working with classmates or colleagues” and “to get others to take you, your ideas or work seriously”. The reasons were derived through reviewing the available camouflaging literature (e.g. Davidson and Henderson 2010; Hull et al. 2017; Tierney et al. 2016) and feedback from two autistic individuals. Internal consistency was good (α = 0.89).

Participants were also asked to share ‘other’ reasons for camouflaging in an open textbox. These qualitative responses were analysed using content analysis. All qualitative responses were reviewed and categorised by two independent raters. The raters met to discuss and agree categories before responses were independently re-coded into the agreed categories, and raters discussed any disagreements before agreeing on final codings for all responses.

#### Camouflaging Contexts

Participants were presented with 22 contexts for camouflaging and rated how often they camouflaged in that context (from ‘never’ (0) to ‘always’ (4)). Example contexts include “with colleagues at work” and “with family members”. These contexts were chosen based around literature on disability disclosure, identity management, camouflaging, and disconnect theory (e.g. Chaudoir and Fisher 2010; Davidson and Henderson 2010; Ragins 2008). The items were reviewed by two autistic individuals and their feedback was incorporated into the measure. Internal consistency was excellent (α = 0.95).

#### Depression, Anxiety and Stress Scale (DASS-21; Lovibond and Lovibond 1995)

Participants rated 21 statements based on their experiences over the past week in terms of depression, anxiety and stress symptoms. Statements were rated from ‘did not apply to me at all’ (0) to ‘applied to me very much or most of the time’ (3). Example items included “I was unable to become enthusiastic about anything”. Seven items each pertained to subscales looking at symptoms of depression, anxiety and stress. Scores were summed for each subscale and multiplied by two, with a possible range of 0–42. Internal consistency was excellent (α = 0.93).

#### Ritvo Autism and Asperger Diagnostic Scale (RAADS-14, Eriksson et al. 2013)

To confirm self-reported diagnoses of autism, participants completed the RAADS-14, a 14-item screening tool which reflects the diagnostic criteria for autism. Example items included “When I feel overwhelmed by my senses, I have to isolate myself to shut them down”. Statements were rated as ‘never true’ (0), ‘true only when I was younger than 16’ (1), ‘true only now’ (2) and ‘true now and when I was young’ (3). A total score is achieved from summing responses, and scores ranged from 0 to 42, with a cut-off score of 14. Internal consistency for this measure was good (α = 0.75).

#### Demographic Questions

Finally, participants reported a number of different demographic characteristics such as age, gender, employment status, sexual identity, education and ethnicity.

### Design and Data Analysis

This study had a cross-sectional survey design. To examine the first hypothesis, that camouflaging disconnection would have a negative impact on psychological wellbeing, camouflaging contexts were analysed using Principal Components Analysis (PCA) to identify overarching categories of contexts (see Results). PCA was utilised because it allowed for the optimisation, reduction and combination of an array of contexts (and reasons) for camouflaging into components which could then be analysed within subsequent analyses. Prorated mean scores for the contexts were used to take into account items which are not applicable to some participants e.g. “With teachers at my child’s school”. Multivariate Analysis of Covariance (MANCOVA) with depression, stress and anxiety scores as the dependent variables was then used to test the impact of camouflaging disconnection, controlling for age, gender and age of diagnosis. All assumptions for parametric data analyses were met.

To test the hypothesis that there would be gender differences in reasons for camouflaging, the reasons were analysed using PCA to test for communalities in the reasons (see Results). As such, Analysis of Covariance (ANCOVA, controlling for current age) was then used to test for gender differences in the broad categories identified as camouflaging reasons, as well as examining interactions with age of diagnosis. A prorated mean score was calculated for the camouflaging reasons, to give a mean score of all of the items that were applicable to the participant—for example, some items such as “To perform well at your job or at university” would not be applicable if a participant was not currently employed or at university.

## Results

Means and standard deviations for the CAT-Q and DASS-21 sub-scales are presented in Table 2, including means according to gender.

**Table 2:** Mean (*SD*) scores for camouflaging (CAT-Q) and depression, anxiety and stress (DASS-21), overall and according to gender

	Total mean (*SD*)	Female mean (*SD*)	Male mean (*SD*)	*p*
Camouflaging score	116.12 (20.48)	118.90 (18.83)	114.25 (21.36)	0.13
Depression	19.68 (10.99)	19.31 (11.02)	20.18 (11.07)	0.54
Anxiety	15.53 (9.64)	15.89 (9.56)	15.26 (10.04)	0.62
Stress	24.06 (9.25)	25.01 (9.48)	22.97 (8.70)	0.084

### Camouflaging Disconnection and Psychological Well-Being

PCA using direct oblimin on the 22 items pertaining to camouflaging contexts identified two camouflaging contexts. The KMO statistic was ‘good’ (0.77; Field 2013) and Bartlett’s test of sphericity was significant (*χ*^2^(231) = 415.39, *p* < 0.001), indicating that correlations between items were large enough to warrant PCA. Table 3 shows the significant item loadings for the two components. The two components explained 50.24% of the variance. The first component was classified as ‘formal’ contexts such as with work colleagues or medical professionals. The second component was classified as ‘interpersonal’ contexts, where the interactions would be more personal such as with friends or family.

**Table 3:** Item loadings for the two extracted components for camouflaging contexts

Item	Formal contexts	Interpersonal contexts
With your university’s administration	0.892	
With your landlord	0.843	
With your bank representative	0.820	
With customer service professionals	0.730	
With someone you’ve just met	0.694	
With students you interact directly with in class	0.670	
With your neighbours	0.648	
With an interviewer or company when applying for job	0.642	
With non-autistic people generally	0.630	
With your boss at work	0.626	
With teachers at my child’s school	0.577	
With doctors or medical professionals	0.547	
With colleagues at work	0.526	
With fellow students generally on campus at university or at school	0.485	
With my child’s friend’s parents	0.437	
With friends		0.816
With your flatmates		0.719
With a romantic or potential romantic partner		0.666
With your Facebook or other social media friends		0.652
With other members of the autism community		0.561
With acquaintances		0.491
With family members		0.437

For each component (formal or interpersonal), the prorated mean rating was computed for each participant. For these two contexts, participants were then categorised as ‘high’ (scoring above the median for formal (3.69) or interpersonal (2.71) contexts) or ‘low’ (scoring below the median) camouflagers for each context. Next, participants were categorised as either consistently high camouflagers (camouflaging rate high in both formal and interpersonal contexts), ‘switchers’ (camouflaging high in one context but low in other) or consistently low camouflagers (camouflaging low in both contexts). CAT-Q scores were used to validate the three groups: ANOVA showed a significant main effect of group (F (2, 180) = 12.03, *p* < 0.001, ηp^2^ = 0.12) and simple effects analyses using Bonferonni found that the low camouflagers’ CAT-Q score (M = 106.08, *SD* = 22.61) was significantly lower than both switchers (M = 118.77, *SD* = 16.19; *p* = 0.002) and high camouflagers (M = 122.63, *SD* = 18.34; *p* < 0.001) and there was no significant difference between switchers and high camouflagers CAT-Q score (*p* = 0.73).

A MANCOVA, controlling for current age, gender and age of diagnosis, tested for differences between the three groups of ‘camouflagers’ (consistent low (*n* = 68), switchers (*n* = 78) or consistent high (*n* = 78)) in terms of depression, anxiety and stress scores from the DASS-21.

Using Pillai’s Trace, there was a significant main effect of camouflage group on mental health, *V* = 0.057, F(6, 430) = 2.12, *p* = 0.05, ηp^2^ = 0.029. No other effects, including the covariates of age, gender and age of diagnosis, were significant (all *p*s > 0.08). Subsequently, separate univariate ANOVAs on the outcome variables showed a significant main effect of camouflage group on anxiety (F(2, 216) = 3.79, *p* = 0.024, ηp^2^ = 0.034) and stress (F(2,216) = 6.23, *p* = 0.002, ηp^2^ = 0.054). There was no significant main effect for depression scores (*p* = 0.14).

**Fig. 1:**
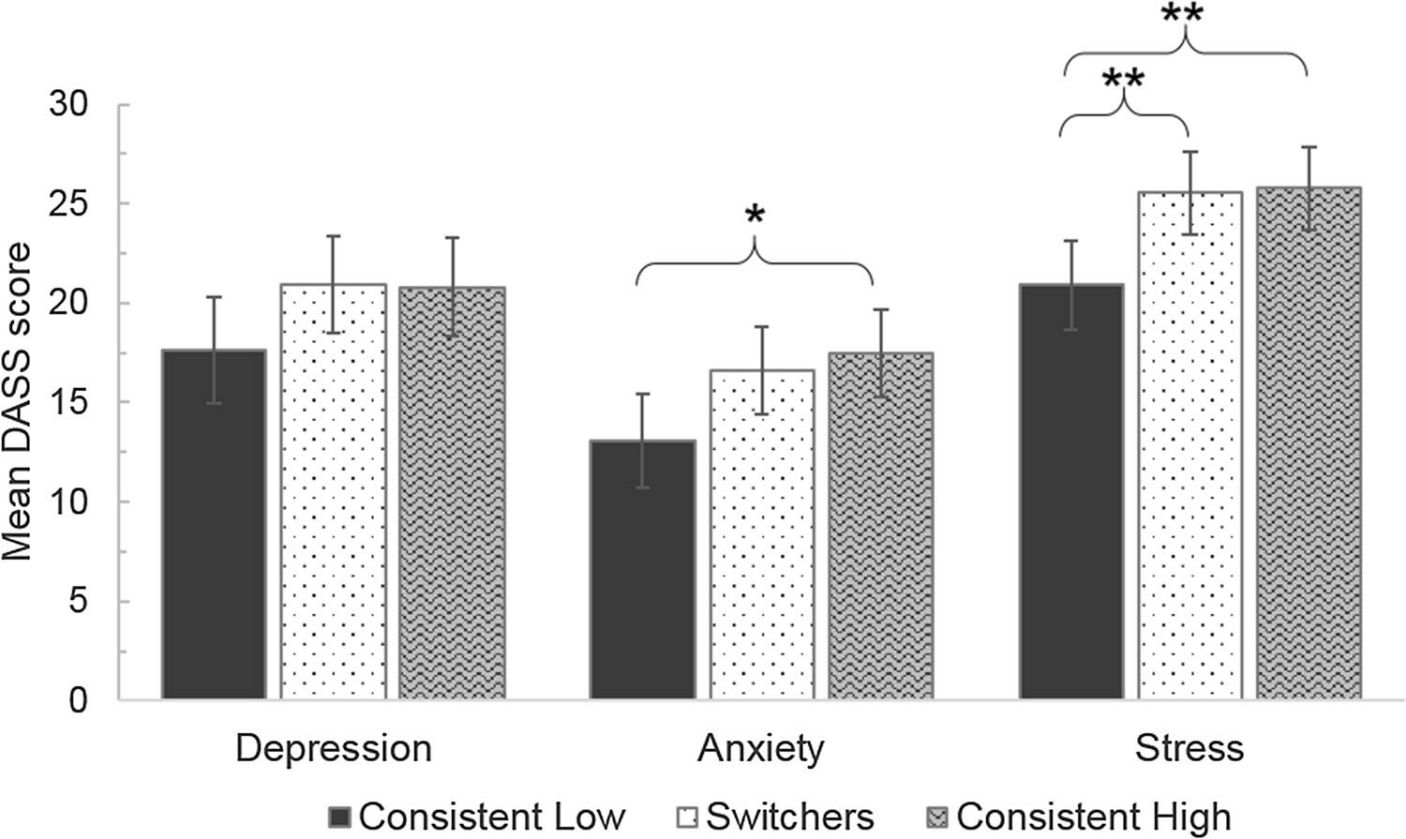
Depression, Anxiety and Stress scores for each camouflage group. **p < 0.01, *p < 0.05. *Note* Error bars +/− 2SE

Simple effects analysis adjusting for multiple comparisons using Bonferroni showed that for stress, those who were consistently low camouflagers had significantly lower stress scores than both switchers (*p* = 0.007) and high camouflagers (*p* = 0.006). For anxiety, there was only a significant difference between those who were consistently low and those who were consistently high (*p* = 0.030), with the low camouflagers showing less anxiety (Fig. 1).

### Gender Differences in the Reasons for Camouflaging

PCA was conducted on the 21 reasons using direct oblimin. The Kaiser–Meyer–Olkin (KMO) statistic confirmed that sampling adequacy was ‘great’ (0.86; Field 2013) and Bartlett’s test of sphericity was significant (χ^2^(210) = 1301.68, *p* < 0.001). Two components were extracted from the data. Item loadings greater than 0.40 were considered to load significantly onto the respective components (Field 2013). Table 4 shows the component loadings. The two components explained 41.04% of the variance. Items clustering on the first component were categorised as ‘conventional reasons’—reasons for camouflaging which appear to serve a primarily functional purpose such as in workplace or educational contexts. The second component was categorised as ‘relational reasons’—reasons for camouflaging which serve to ease everyday social interactions and relationships.

**Table 4:** Item loadings for the two extracted components for camouflaging reasons

Item	Conventional reasons	Relational reasons
To communicate your ideas or work	0.831	
To perform well at your job or at university	0.791	
To aid working with classmates or colleagues	0.736	
To get others to take you, your ideas, or work seriously	0.731	
To get a job	0.607	
To reduce awkwardness in social interactions	0.551	
To impress your superiors at work or lecturers at university	0.511	
To demonstrate that I am a responsible person	0.477	
To get a promotion	0.449	
To make friends		0.796
To seem attractive to a potential romantic partner		0.750
To appear likeable		0.700
To bond with others		0.684
To fit in with others		0.568
To demonstrate my successes		0.543
To express my trustworthiness		0.425
To express my intelligence		0.402

*Note* Four items did not load onto the two components: ‘To reduce stigma, stereotypes or discrimination against you’; ‘Because it is expected of you’; ‘To find a flat or house to live’ and ‘To make others feel more comfortable’

Following this PCA, a prorated mean score for each participant was calculated for the items corresponding to conventional reasons and relational reasons respectively. To test for gender differences in camouflaging reasons, these means were used as the dependent variable in a two (reason: conventional or relational) by two (gender: male or female) by three (diagnosis: childhood, early adulthood (18–34) or later adulthood (35–64)) mixed design Analysis of Covariance (ANCOVA), controlling for age. All assumptions for the model were met.

**Fig. 2:**
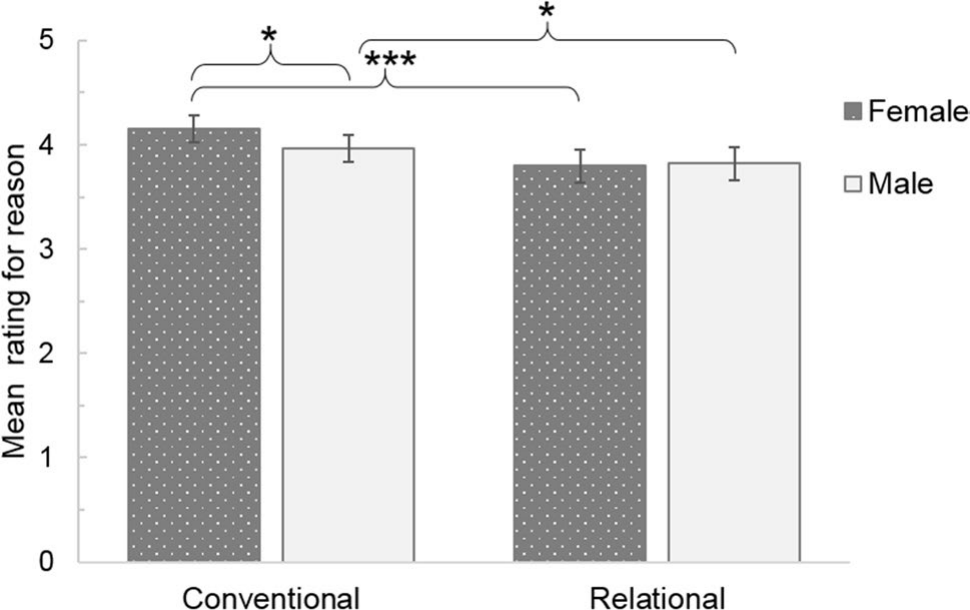
Mean ratings for conventional and relational reasons for males and females. *p < 0.05; ***p < 0.001. *Note* Error bars +/− 2SE

There were no significant main effects (all *p*s > 0.15). There was a significant interaction between reasons and gender, F(1, 215) = 5.16, *p* = 0.024, ηp^2^ = 0.023. Simple effects analyses using Bonferroni to correct for multiple comparisons showed a significant difference between males and females for conventional reasons (*p* = 0.043) but not relational reasons (*p* = .84; Fig. 2), with female participants endorsing conventional reasons more than male participants. Further, both female participants (*p* < 0.001) and male participants (*p* = 0.034) rated conventional reasons more highly than relational reasons.

**Fig. 3:**
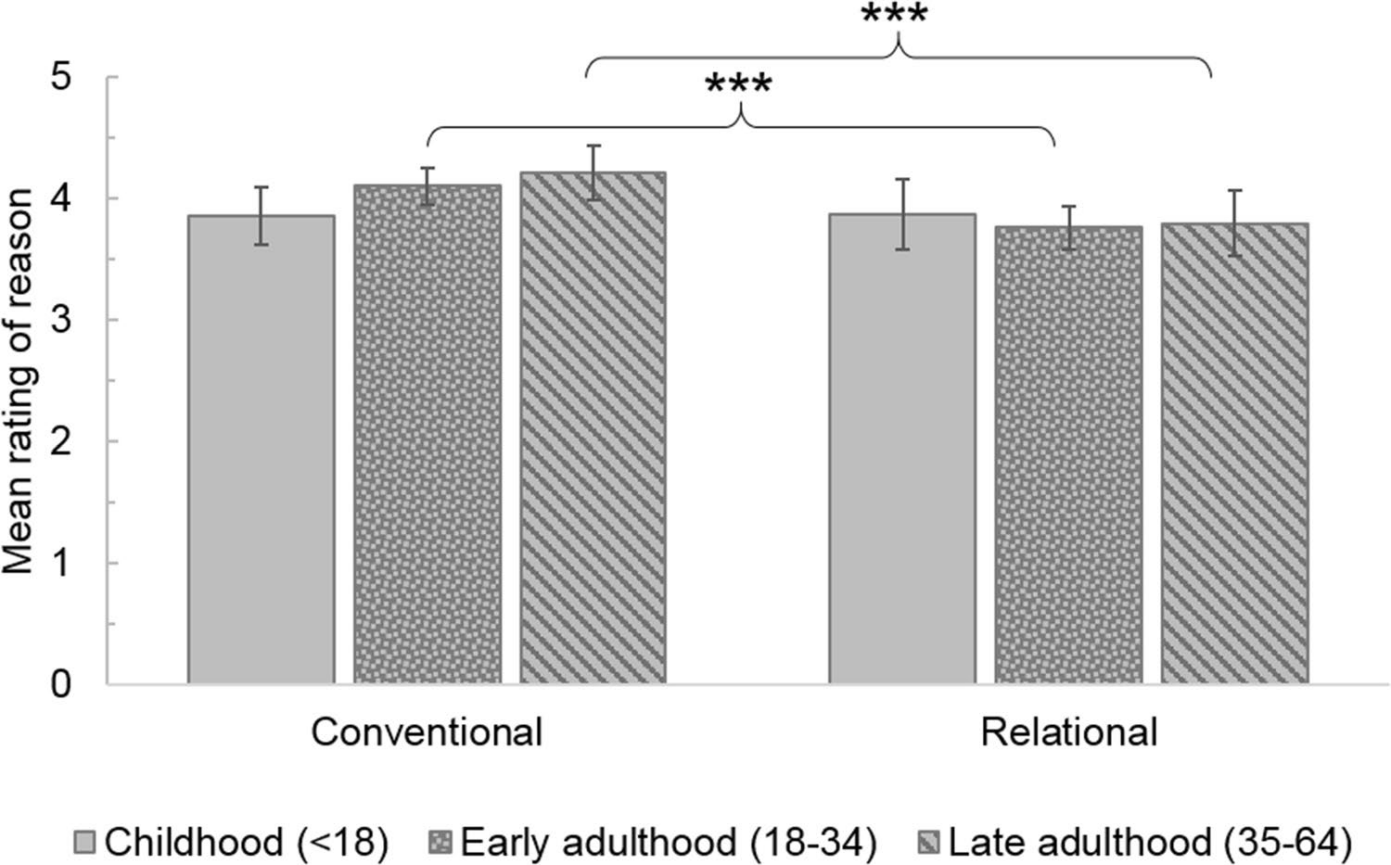
Mean ratings for conventional and relational reasons for age of diagnosis groups. ***p < 0.001. *Note*: Error bars +/− 2SE

There was a significant interaction between reasons and age of diagnosis, F(2, 215) = 4.33, *p* = 0.014, ηp^2^ = 0.039. All other interactions were not significant (*p*s > 0.14). Simple effects analysis with Bonferroni was used to examine the interaction between reasons and age of diagnosis: both those diagnosed in early and later adulthood rated conventional reasons more highly than relational reasons (both *p*s < 0.001), however there was no difference in these ratings for those diagnosed in childhood (*p* = .93; Fig. 3). There were no significant differences between diagnosis groups in their ratings (*p*s > 0.11).

### Qualitative Findings: Reasons for Camouflaging

Content analysis was used to interpret responses given when participants were asked for ‘other’ reasons for camouflaging. 91 participants gave responses. Five themes were identified and agreed upon. Themes and sample quotes are shown in Table 5.

**Table 5:** Themes identified for ‘other’ camouflaging reasons, with *n* and sample quotes

Theme	*N*	Example quotes
Fitting in and passing in a neurotypical world	42	“Because society expects you to behave like neurotypical people” “To get through situations as painlessly and as quickly as possible”
Avoiding retaliation and bullying by others	34	“To protect myself from violence, intimidation, bullying and harassment which happen more when I am “out” as autistic than when they don’t know” “To stop bullying and mocking as I’ve experienced this when not masking”
Concerns about impression made when not camouflaging	32	“Because it makes my wife less embarrassed to be seen with me” “As a parent, to show I’m competent in front of other parents/ to teachers”
Habit	16	“A lifetime of conditioning, trained to act normal/not being normal was bad as a child and now it seems impossible to turn it off” “I’ve been doing it for so long it’s become a habit. I prefer not to have to do it, but to some extent it’s become a protective thing and I feel vulnerable not masking”
Internalised stigma	12	“To avoid feeling ashamed” “Being me isn’t good enough”

The most common theme was ‘*Fitting in and passing in a neurotypical world*’ (*n* = 42). This theme characterised responses which indicated that camouflaging was driven by a desire to assimilate or ‘pass’ within neurotypical society, to not stand out or feel different in comparison to others, and to be able to fit into non-autistic social groups. The second most common theme was ‘*Avoiding retaliation and bullying by others*’ (*n* = 34). This theme was characterised by statements expressing that the reason for camouflaging was to avoid adverse or negative reactions from others when disclosing their autistic identity. Often, participants referred to specific past experiences that had led them to using camouflaging as a strategy to protect against future retaliation, with camouflaging helping them to feel safe or protected against negative reactions.

The third most common theme was ‘*Concerns about impression made when not camouflaging*’ (*n* = 32). This theme identified that some participants used camouflaging as a tool to manage others’ impressions by presenting a particular image of the self, for example to demonstrate their competence and skills, or to avoid others feeling embarrassed or uncomfortable when they were not camouflaging. Two other less common themes were identified: ‘*Habit*’, that some (*n* = 16) explained that they camouflaged not out of choice, but because camouflaging had become a habitual and automatic response in social situations. Finally, the theme of ‘*Internalised stigma*’ was identified, with some participants (*n* = 12) reporting that the reason that they camouflaged was because they felt a sense of shame surrounding their identity.

## Discussion

The current study examined reasons for camouflaging, different contexts for camouflaging, and costs of camouflaging for mental health. Using Disconnect Theory (Ragins 2008), two contexts for camouflaging, formal and interpersonal contexts, were identified. Participants who switched between camouflaging in one context but not the other showed equivalent anxiety and stress symptoms to those who camouflaged highly in both contexts. Those who were classified as ‘consistently low’ camouflagers had significantly lower stress symptoms than both switchers and high camouflagers, and significantly lower anxiety symptoms than high camouflagers. Camouflaging, as such, appears to be costly in terms of stress and anxiety, and camouflaging in some situations but not in others could be as costly as camouflaging all of the time. Two reasons for camouflaging were also identified: ‘conventional’ reasons (to get by in formal settings like work or education) and ‘relational’ reasons (to get by in relationships with others). Autistic women were more likely to endorse ‘conventional’ reasons than men. Qualitative responses also added that participants camouflaged to ‘pass’ in the neurotypical world, to avoid bullying or retaliation and to manage other’s impressions of them.

In terms of contexts for camouflaging, the current findings partly follow Disconnect Theory’s (Ragins 2008) expectations that “switchers” will have high levels of psychological distress. Both switchers and high camouflagers showed significantly higher ratings of stress symptoms in comparison to low camouflagers. This finding suggests that disconnection could produce as much psychological strain in the form of stress as consistently high rates of camouflaging. These equivalent levels of stress could be the result of disguising one’s identity across contexts (Ragins et al. 2007). The results for high camouflagers fall in line with previous literature correlating mental health symptoms with camouflaging (Cage et al. 2018a; Hull et al. 2017). While those who switch are less impacted by constantly hiding their identity, they are nonetheless burdened with expending energy evaluating the perceived risk of exposing their autistic identity in each context. This constant self-regulation may therefore bring them to the same level of stress as those consistently camouflaging. As this is the first instance of Disconnect Theory (Ragins 2008) being applied to camouflaging in autism, further research is warranted to examine the impact of this disconnection in more depth.

With anxiety symptoms, switchers were not different to either low or high camouflagers. High camouflagers did however show significantly higher anxiety symptoms than low camouflagers. One possibility could be that high camouflagers show increased anxiety due to a constant strain of camouflaging—unlike the ‘switchers’ or low camouflagers, they have fewer opportunities to ‘take the mask off’. This finding fits with previous qualitative research whereby autistic people have discussed the experience of anxiety after camouflaging (Hull et al. 2017). This anxiety could also reflect the lack of a safe space to exhibit one’s full identity, another common experience noted in qualitative research (Bargiela et al. 2016; Hull et al. 2017). However, another possibility is that high camouflagers are camouflaging in response to high levels of social anxiety. Social anxiety is common in autistic adults (Spain et al. 2018) with social anxiety characterised by the avoidance of social situations in addition to cognitive features like fear of negative evaluation (Maddox and White 2015). The potential relationship and directionality between social anxiety and camouflaging behaviour requires further investigation.

Interestingly, there was no difference between the three groups of camouflagers in terms of depression. It should be noted that the levels of depression were high, particularly in comparison to non-autistic population scores (a mean score of 19.68 in comparison to 5.66 in Henry and Crawford (2005)). This finding conflicts with Cage et al.’s (2018a) finding of higher depressive symptoms, but not anxiety or stress, in those who camouflaged compared to those who did not. However, this discrepancy in findings may be due to Cage et al.’s (2018a) study using participants’ spontaneous reports of camouflaging, which potentially excluded people who camouflaged but did not explicitly report it. The relationship between camouflaging and depression requires further investigation, especially given the relationships between depression and suicidality, and the links between camouflaging and suicidality (Cassidy et al. 2018).

The current study also found gender differences in the reasons for camouflaging. Specifically, findings indicated that autistic women endorsed ‘conventional reasons’ more highly than males. These conventional reasons centred on camouflaging to get by in work or education, such as to aid working with colleagues or classmates. There were no gender differences in endorsement of relational reasons, such as camouflaging to make friends or fit in with others. It should be noted that both men and women endorsed conventional reasons more than relational reasons, but women endorsed conventional reasons *more* than men. These findings could be explained through an intersectional approach to camouflaging. Intersectionality would argue that autistic women face specific barriers enforced by the male-dominated narrative around autism (Saxe 2017). The expectations (or lack of understanding) placed on autistic women in conventional settings—like the workplace or at university—may mean that they feel that camouflaging is needed more in those settings. Indeed, in Bargiela et al.’s (2016) interview study, late-diagnosed women discussed how they struggled to fit in with societal expectations around gender roles (such as being a mother or girlfriend). This interpretation is further supported by a recent study by Botha and Frost (2018), which found that many autistic individuals, much like other minorities related to race, sexuality or religion, are subject to the minority stress model (Altman 2001; Smart 2006), whereby everyday discrimination and internalised stigma lay the groundwork for poor mental health (Botha and Frost 2018). As women are often a marginalised minority with minimised social standing, autistic women have multiple minority statuses, which may have contributed to the results seen in the present study. It is important that society’s role in enforcing stereotypes both around women and autism is not ignored when it comes to understanding camouflaging.

Age of diagnosis (irrespective of gender) also interacted with the reasons for camouflaging, with those diagnosed in adulthood endorsing conventional reasons more than relational reasons. Those diagnosed in childhood did not differ in their ratings of conventional and relational reasons. It may be that this finding is impacted by autistic people’s experiences prior to receiving a diagnosis: they will have spent more time navigating situations without a diagnostic label when it could have been beneficial for receiving support such as at school (Jones et al. 2014). Receiving confirmation of being autistic can be a validating experience, although lack of post-diagnostic support is of paramount concern (Crane et al. 2018). More research is needed to examine the potential differences between camouflaging pre- and post-diagnosis.

Importantly, qualitative findings here add depth to understanding the reasons for camouflaging. The most frequently reported reason was that camouflaging was used to ‘pass’ or fit into neurotypical society, which conforms with the definition of camouflaging (Hull et al. 2017). The second most reported theme was that camouflaging helped autistic people avoid bullying and retaliation. Autistic individuals are frequently targeted by bullies (Schroeder et al. 2014), with estimates suggesting autistic individuals are four times more likely to have been bullied than neurotypical individuals (Sterzing et al. 2012). It is worth considering how, despite the costs of camouflaging on mental health, autistic people must weigh up the costs of bullying and non-acceptance when not camouflaging. Accordingly, camouflaging could be a response to stigmatisation: to protect and manage an identity which is stigmatised by others, camouflaging may be used as a protective strategy.

Indeed, previous studies have explored how autistic individuals often report experiences of stigma (Shtayermman 2009), misunderstandings and underestimation of their abilities (Heasman and Gillespie 2017) as well as neurotypical people generating more negative judgements in first impressions (Sasson et al. 2017) and dehumanising them (Cage et al. 2018b). These findings suggest that autistic people encounter a ‘double empathy problem’ (Milton 2012), such that autistic people struggle to understand the social intricacies of the neurotypical world, but neurotypical people also struggle to understand autistic people’s sociality. With the high rates of camouflaging reported in autistic people, as noted here and elsewhere (e.g. Hull et al. 2017), it appears autistic people invest a significant amount of time and energy into understanding and trying to fit in to the neurotypical world (often to the detriment of their mental health), rather than neurotypical people attempting to understand autistic people’s world and adapt accordingly. Given the potential impact of non-acceptance on the mental health of autistic people (Cage et al. 2018a), it is vital that more research on improving non-autistic people’s attitudes towards autism is conducted.

### Limitations and Future Directions

The current study is not without limitations. Although there was a relatively large sample size, the sample was poorly represented in terms of ethnic diversity and educational status, with mostly White participants who had received Higher Education. It may be argued that the sample consisted of highly verbal individuals, which would not be representative of autistic individuals with additional learning disabilities. Unfortunately, much of autism research is limited in its generalisability in this way (Pellicano et al. 2014) and researchers should endeavour to examine more diverse communities. Nonetheless, there is also a paucity of research specifically on the experiences of autistic adults (Pellicano et al. 2014), therefore the current study does add to a growing body of literature focusing on life beyond childhood.

One pertinent issue that the current study has raised focuses on the need to improve acceptance and reduce stigmatisation by non-autistic individuals. Preliminary evidence has been found on anti-stigma interventions focused on improving acceptance of autistic girls in schools (Ranson and Byrne 2014). Gillespie-Lynch et al. (2015) found that after participating in online training about autism, university students showed more autism knowledge and less stigma. More research is needed with non-student populations, as well as long-term educational interventions to test for reduced stigma over time. Further, the variable of experience or contact with autistic people often relates to more positive attitudes towards autism (e.g. Gillespie-Lynch et al. 2015; Nevill and White 2011; White et al. 2016). It is therefore important that autistic voices are heard within any interventions.

This study also has several clinical implications. First, in terms of diagnosis of autism, particularly for women and those seeking diagnosis in adulthood, clinicians should be aware of the presence of camouflaging behaviours. Since the gender ratio in diagnosis of autism has recently been suggested to be 3:1 (males to females), rather than the previously supposed 4:1 (Loomes et al. 2017), this suggests that clinicians may be improving at recognising autism in girls and women. Clinicians must remain aware of the societal and gendered expectations that could cloud diagnostic judgements. Second, when treating the comorbid mental health conditions experienced by autistic people, it would be important for the clinician to discuss whether camouflaging is impacting on the individual’s psychological wellbeing, and if so, support the individual to identify strategies to reduce camouflaging. It is important to note, however, that appropriate support services for autistic adults are thought to be lacking (Turcotte et al. 2016), with a drastic need for evidence-based autism-specific mental health interventions (Murphy et al. 2016; Shattuck et al. 2012), therefore autistic people may have limited opportunities to receive appropriate support for their mental health.

Further, clinicians should understand the ways in which camouflaging can be a maladaptive strategy, given the significant costs to psychological wellbeing that have been identified. It might be argued that camouflaging has some adaptive benefit, for example to help navigate new environments or, as mentioned in the qualitative responses in the current research, to simply “get through situations as painlessly and as quickly as possible”. Both autistic and non-autistic people may use self-presentational strategies to make impressions on others and to navigate social situations (Cage et al. 2013; Scheeren et al. 2016). However, for autistic people, the potentially adaptive aspects of camouflaging ultimately reflect the lack of understanding produced by the neurotypical world, and the immense effort that those that do not fit into that world must make in order to “pass”, avoid being bullied, or have their work recognised. Perhaps clinicians could keep in mind methods of support that help autistic people to succeed as autistic people, rather than autistic people masquerading as neurotypicals.

## Conclusion

The current study enhances understanding of camouflaging in autism through demonstrating the potentially harmful effects of camouflaging on mental health, especially for those who report high rates of camouflaging and those who inconsistently camouflage in different situations. Further, this study adds to the debate surrounding the role of camouflaging for autistic men and women (Lai et al. 2017), suggesting that camouflaging occurs for both genders, but autistic women potentially face additional stigma which may differentially influence their camouflaging behaviour. Camouflaging must, therefore, be viewed not only as a psychological but sociological phenomenon.

## Footnotes

1 This paper uses identity-first language to reflect the preferences of the autism community (Kenny et al. 2016).
